# A comprehensive validation of HBV-related acute-on-chronic liver failure models to assist decision-making in targeted therapeutics

**DOI:** 10.1038/srep33389

**Published:** 2016-09-16

**Authors:** Yi Shen, Xulin Wang, Sheng Zhang, Gang Qin, Yanmei Liu, Yihua Lu, Feng Liang, Xun Zhuang

**Affiliations:** 1 Department of Epidemiology and Medical Statistics, Nantong University, Nantong, China; 2 Center for Liver Diseases, Nantong Third People’s Hospital, Nantong University, Nantong, China; 3 Qidong Third People’s Hospital, Nantong, China

## Abstract

This research utilized an external longitudinal dataset of hepatitis B virus-related acute-on-chronic liver failure (HBV-ACLF) to compare and validate various predictive models that support the current recommendations to select the most effective predictive risk models to estimate short- and long-term mortality and facilitate decision-making about preferable therapeutics for HBV-ACLF patients. Twelve ACLF prognostic models were developed after a systematic literature search using the longitudinal data of 232 HBV-ACLF patients on the waiting list for liver transplantation (LT). Four statistical measures, the constant (A) and slope (B) of the fitted line, the area under the curve (C) and the net benefit (D), were calculated to assess and compare the calibration, discrimination and clinical usefulness of the 12 predictive models. According to the model calibration and discrimination, the logistic regression models (LRM2) and the United Kingdom model of end-stage liver disease(UKELD) were selected as the best predictive models for both 3-month and 5-year outcomes. The decision curve summarizes the benefits of intervention relative to the costs of unnecessary treatment. After the comprehensive validation and comparison of the currently used models, LRM2 was confirmed as a markedly effective prognostic model for LT-free HBV-ACLF patients for assisting targeted and standardized therapeutic decisions.

Caused by the acute exacerbation of chronic hepatitis B (CHB), hepatitis B virus-related acute-on-chronic liver failure (HBV-ACLF) is a severe life-threatening disease in patients who have previously diagnosed or undiagnosed chronic liver disease^1^,^2^. In Asia, there is a high prevalence of HBV in developing countries where HBV-ACLF accounts for more than 70% of ACLF and almost 120,000 patients die of HBV-ACLF annually^3^,^4^. Provided that liver transplantation (LT) is not arranged in time, ACLF patients have a poor prognosis with short-term mortality ranging from 30% to 70%^5^.

Because donor livers are often not available in time, the development of an artificial liver support system (ALSS) plays an important role in the bridge to LT. Our previous study reported that the 90-day and 5-year mortality rates in the ALSS group were significantly lower than in the control group (40% vs 53%, and 57% vs 69%, respectively)^6^. However, the overall efficacy of ALSS has failed to reach a level sufficient to gain approval for widespread use^7^. To guide and optimize targeted therapeutics in HBV-ACLF patients on the waiting list for LT, a proper and accurate prognostic scoring system is urgently needed to better assess risk and help physicians decide whether to initiate ALSS therapy or to choose conservative treatment. During the past two decades, a large number of prediction models have been developed to assess liver function, such as the end-stage liver disease system, including a model of end-stage liver disease (MELD)^8^, a sodium MELD (MELD-Na)^9^,^10^, a MELD to sodium ratio (MESO)^11^, an integrated MELD (iMELD)^12^,^13^, an updated MELD (uMELD)^14^, the United Kingdom MELD (UKELD)^15^ and a donor MELD (D-MELD)^16^; as well as the Child-Turcotte-Pugh class (CTP) based system, including CTP^17^ and modified CTP (mCTP)^18^. Recently, several logistic regression models (LRMs) were adopted to predict the survival rates of Chinese ACLF patients^19^,^20^.

The mortality risk expected for similar patients is a significant component in targeted intervention. Therefore, a direct comparison of the performance of existing models in the same external population is essential for bridging the gap between developing models and designing studies for clinical utility. In general, few studies have validated ACLF models externally, no more than two or three studies exist and almost all were conducted in short-term survival cohorts. In addition, three recent reviews regarding this topic have described standard MELD validation in advanced cirrhosis or ACLF patients compared to other MELD-based models^21^,^22^,^23^. CTP-based and LRM-based systems have never been externally validated. Meanwhile, traditional comparative approaches consider only the predictive discrimination of models. Recently, a number of decision–analytic measures have been proposed to assess the clinical usefulness of models, such as the use of “decision curves” to plot the net benefit achieved by making personalized decisions on the basis of model prediction^24^.

The objective of this study is to employ an external longitudinal dataset of HBV-ACLF patients to compare and validate various predictive models supporting the current recommendations in order to select the most effective predictive risk models to estimate short- and long-term mortality risk and facilitate decision-making about preferable therapeutics for LT-free patients. Our research consists of two parts: (a) a systematic review conducted to identify relevant existing models for predicting the future risk of ACLF patients and (b) various statistical measures adopted to validate and compare the prognostic performance of different models in external longitudinal data and to choose the best model to assist clinical decision making for HBV-ACLF patients.

## Results

### Systematic literature search

**Figure 1:**
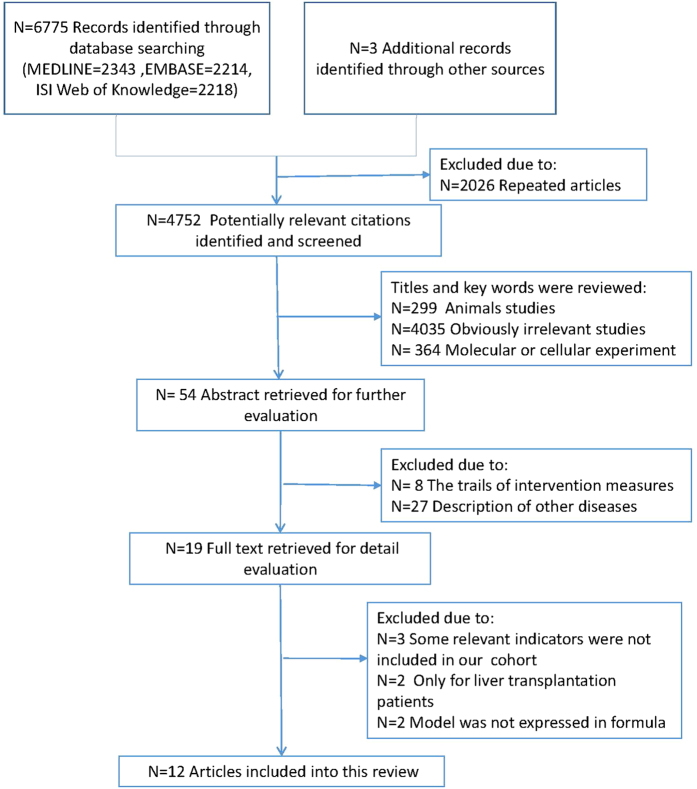
Study selection flow diagram. An overview of the systematic literature search of studies that derived prediction models for ACLF. ACLF: acute-on-chronic liver failure.

A total of 4752 articles were identified through an online database search according to our keyword strategy (Fig. 1). After reviewing the titles and abstracts, 4698 and 35 studies were excluded, respectively. Among the remaining 19 full-text articles, seven articles^16^,^24^,^25^,^26^,^27^,^28^,^29^ were excluded for the absence of relevant indicators or because they contained explicit mathematical expressions in their models. Twelve models were eventually included in our study: eight were in the MELD-based system^8^,^9^,^10^,^11^,^12^,^13^,^14^,^15^, two were in the CTP-based system^17^,^18^ and two were in the LRM-based system^19^,^20^. Table 1 summarizes the characteristics of the 12 models included in this validation study are summarized. Table S1 presents the prognostic indicators of these models. In addition, 3-month and 5-year hazard ratios of all models were significant using *Cox* proportional hazards model.

### Calibration analysis of the twelve models

Calibration plots for the twelve predictive models are shown in Figure S1 and Figure S2, and the intercepts (A) and slopes (B) of the fitted lines are shown in Table 2. For A at 3 months, most models had well-calibrated interception values, which ranged from 0.118 to 1.429, except for MELD (A = 3.689). The sorted sequence of absolute distance from 0 was LRM1, iMELD2, LRM2, mCTP, MELD-Na1, MELD-Na2, CTP, uMELD, iMELD1, MESO, UKELD and MELD. However, the sorted sequence of absolute distance from 0 at 5 years was different from that at 3 months: UKELD, MELD-Na2, MELD, uMELD, LRM2, MELD-Na1, LRM1, iMELD2, MESO, iMELD1, CTP and mCTP.

For B, all slope values were in close proximity to 1 and showed good consistency for all models at 3 months and 5 years. At the 3-month time point, all models overestimated the predicted risk compared to the observed predicted risk of ACLF, except for iMELD2, LRM2 and MELD-Na2. At the 5-year time point, MELD, MELD-Na2, iMELD1, iMELD2, MESO, uMELD, LRM1 and LRM2 overestimated the predicted risk of ACLF.

### Discrimination analysis of the twelve models

In Table 2, the C values ranged between 0.72 and 0.82, which proved that allmodels except for MELD, MESO and MELD-Na2 performed well in terms of their discrimination at 3 months. A slightly higher C range from 0.74 to 0.85 was observed for the 5-year risk prediction for all models except MELD. With the highest C value at 3 months and 5 years, LRM2 exhibited the best diagnostic accuracy, followed by UKELD and uMELD at 3 months and UKELD and iMELD1 at 5 years. The 3-month and 5-year ROC curves are displayed in Figure S2. According to the optimal cut-off point, the sensitivity, specificity, positive predictive value (PPV) and negative predictive value (NPV) of every model are reported in Table S3.

### The decision curve analysis of the twelve models

**Figure 2:**
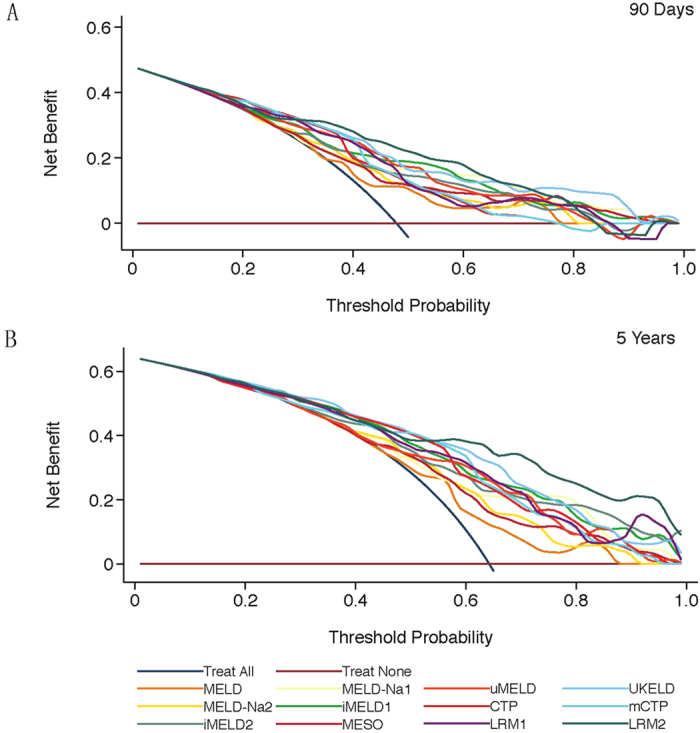
Decision curves for the prediction models applied in longitudinal data (**A**) 90 days, (**B**) 5 years. MELD: model of end-stage liver disease; MELD-Na: sodium MELD; MESO: MELD to sodium ratio; iMELD: integrated MELD; uMELD: updated MELD; UKELD: United Kingdom MELD; CTP: Child-Turcotte-Pugh class; mCTP: modified CTP; LRM: logistic regression model.

Figure 2 demonstrates the decision curves for the 12 models in predicting 3-month and 5-year mortality. A straight line was drawn on the bottom to show the outcomes without treatment (i.e., no net benefit). A navy smooth curve was drawn as if all patients received treatment, irrespective of their prognostic results. Figure 2a shows that the LRM2 curve is superior to the others, with a wide interval between 30% and 75%. The MELD curve was very close to the navy curve for “treat all” and the other curves overlapped with each other and demonstrated only small differences in the net benefit between 30% and 75%. In Fig. 2b of the 5-year curves, the LRM2 curve was best and possessed the widest interval of the curves, and the MELD curve was close to the “treat all” curve.

According to a survival meta-analysis of HBV-ACLF patients, 40% *p*_t_ was substituted into the formula above to compute the 3-month net benefit (D) of the 12 models^30^. As shown in Table 2, LRM2, UKELD and mCTP were the best three models for the 3-month prognosis. However, there were no meta-analyses for the long-term survival of HBV-ACLF, although 55% *p*_t_ was extracted in one article^6^. LRM2, UKELD and mCTP were the three best models for the 5-year prognosis, which presented similar arrangements as the 3-month models.

## Discussion

HBV-ACLF is caused by spontaneous severe acute exacerbation in patients with CHB, which results in jaundice, coagulopathy or hepatic encephalopathy and sometimes liver failure, with high short-term (28 days) (>15%) and annual mortality rates(>50%)^1^,^31^. The importance of the diagnosis and prognosis for impending organ failure cannot be overemphasized in this particular group of patients, as a timely intervention can prevent or reverse the process and improve survival. For HBV-ACLF, ALSS is a useful approach for replacing liver function by granting a chance for hepatic recovery or through stabilizing the clinical state to accomplish transplantation^32^. However, the cost-effectiveness and unclear benefits of ALSS lead to no improvement in overall survival. Physician and patient groups have called for a better method to correctly identify patients at high risk of mortality to facilitate indispensable treatment and to avoid the unnecessary burden for low risk patients^33^,^34^.

In the present study, LRM2 was identified as the most validated model for both 3-month and 5-year prognoses according to four key measures: “A, B, C, and D”, which showed reasonable accuracy and improved treatment decision-making. LRM2 was established and validated in internal and external cohorts by Zheng *et al.*^20^. The MELD and CTP scoring systems are used mainly in cases of decompensated cirrhosis. However, HBV-ACLF represents a complex condition that differs from cirrhosis in many respects. To eliminate variation in the objective indicators between different laboratories, instruments, and reagents, LRM2 includes clinical parameters that usually rely on certain empiric predictive variables^35^. Furthermore, as a target antigen for immune elimination, HBeAg is closely associated with immune-mediated liver injury^20^. Based on blood coagulation function, liver cirrhosis, hepatic encephalopathy, hepatorenal syndrome and aetiology (HBeAg), the composition of LRM is more reasonable.

Due to objective and quantitative variables, MELD is the most commonly used model for evaluating patients on the waiting list for LT^36^. However, it lacks indicators of clinical events, such as hyponatremia, hepatorenal syndrome and other complications, which are significantly related to the natural history of viral hepatitis and outcomes in longitudinal studies. In addition, modified MELD-based scores that have incorporated the measurements of age and serum sodium are available to predict liver disease prognosis, such as MELD-Na, iMELD, MESO and UKELD. In the present study, these scores were compared, and UKELD was predictive of mortality risk for LT-free HBV-ACLF patients.

An evaluation of calibration is important if model predictions are used to inform patients or physicians decision-making^37^. In the present study, a value of A more than 0 and a value of B smaller than 1 was common, which demonstrates that the predictions were extreme; the low values tended to be even lower, and the high values tended to be even higher. Compared with the 3-month predictions, this variation was more pronounced for the 5-year outcomes, which reflects relatively higher variability. The C value was applied broadly to account for the fairly artificial classification in a pair of patients who did or did not die. In our study, the majority of basic models focused on the high identification capacity of this external longitudinal data with C values over 0.70.

The “A, B and C” values measured only the calibration and discrimination of models. In fact, a model with much greater specificity but slightly lower sensitivity would have a higher AUC, which would be a poorer clinical choice because a false-negative result is more harmful than a false-positive result^38^. D values were determined by calculating the difference between the expected benefit and expected harm in a simple, parsimonious method for evaluating alternative clinical strategies. In particular, the risk/benefit ratio captured the patient’s value regarding the risks of under-and overtreatment, which could be considered to summarize the benefits of the intervention relative to the costs of unnecessary treatment and to show benefit to a broad range of similar patients.

Interestingly, according to the D value of 40% *p*_t_ at the 3-month time point, mCTP took the place of uMELD among the top three models, although mCTP had a smaller AUC than uMELD (0.74 vs 0.77). Undoubtedly, a sensitive predictor is superior to a specific predictor because *p*_t_ is less than 50%, which means that the harm of a false negative is greater than that of a false positive. Several different parameters of liver disease were considered in the CTP scoring system to display the cirrhosis severity of HBV-ACLF patients, such as ascites and hepatic encephalopathy. With the addition of cirrhotic patients with higher scores awaiting LT in an Asian centre, mCTP was proposed to attenuate the ceiling effects by extending the rating system up to 18 points to further improve the CTP-based system^18^.

Many decision-making indexes, including the net reclassification improvement (NRI), the weighted NRI (wNRI) and the relative utility (RU), have been suggested to evaluate the usefulness of a prediction model in practice, namely by assisting with clinical decisions regarding treatment^39^,^40^,^41^,^42^. Lee *et al*. recently proposed a new index called the APAPT, which was the average deviation about the probability threshold^43^. This index both acknowledges positive outcomes and explains the negative result of predictive models. However, the net benefit was considered a preferable method for the facilitation of clinical decision-making about alternative therapeutics for HBV-ACLF patients. First, it does not require information on the costs or effectiveness of treatment or how patients value different health states; second, the method can be applied to a model validation dataset more graphically and directly than other methods; and third, it refines risk classification, which improves the targeting of individuals who will benefit from therapeutic interventions. Notably, the LRM2 and UKELD were still the best models for both the 3-month and 5-year data when comparing the ADAPT values of the twelve predictive models (not shown), which could be explained by the robust decision-curve analysis.

To the best of our knowledge, this is the first use of a 3-month and 5-year longitudinal study to compare the validation and accuracy of relative score systems as predictors for LT-free patients with HBV-ACLF. In accordance with the most comprehensive systematic literature search of all ACLF models and the most comprehensive statistical validation of the “A, B, C and D” values, LRM2 is the most accurate model for both the 3-month and 5-year prognoses. Although it is known that *logistic regression* requires less power than the *Cox proportional hazard method*, which is the most common analysis of time-to-event data, LRM2 results in the greatest prognostic value because the target population is Chinese HBV-ACLF patients, which is also the population represented in our data. Other scoring systems have been established and validated in developed countries, and most patients were hepatitis C, alcoholic and cholestasis liver disease patients^8^,^9^,^10^,^11^,^12^,^18^. Ethnic differences make the LRM system more applicable in our target subjects.

Nevertheless, there are several limitations to our study. A potential limitation is the exclusion of a number of risk scores as necessary information was not available. A second limitation is the lack of a standard sample size estimation in model validation studies. One study suggests that 100 events and 100 non-events are the minimum samples required for external validation studies^44^. In our study, the ratios of dead to living patients at 3 months and 5 years were 111/121 and 149/83, respectively. The smaller number of patients alive at 5 years is understandable in the final dataset, although this could have affected the model validation procedures. Another limitation is that the follow-up data were collected from a single centre, and only HBV patients were included in the external validation. Our results could not be readily applicable to American or European patients in whom hepatitis C and alcoholism are the predominant causes of end-stage liver diseases or in whom the determining factor for prognosis is the degree of end-organ failure^45^.

Depending on the therapeutic strategy and host factors, disease progression might be subtly variable. A careful discussion with the patient and a decision analysis remain challenging for the elicitation of health state preferences and personalized treatment. Taking these models into consideration is not just a matter of the use of a numerical rating scale to determine characteristics and outcomes but also demonstrates how data are interpreted to formulate policies and to encourage future studies to achieve better survival opportunities for LT-free HBV-ACLF patients.

## Conclusion

LRM2 is confirmed as a markedly valued prognostic model for LT-free HBV-ACLF patients to facilitate decision-making options for targeted therapeutics.

## Materials and Methods

### Systematic literature search

Relevant papers published prior to February, 2014 were identified through a search of the PubMed, Embase, and Web of Science databases using the following terms: (“scoring system” OR “score system” OR “prediction model” OR “predictive model” OR “prognosis model” OR “risk assessment”) AND ((“acute liver failure” OR “fulminant hepatic failure” OR (“acute-on-chronic” AND “liver disease”) OR (“cirrhosis” AND “decompensation”) OR “decompensated cirrhosis” OR (“cirrhosis” AND “acute”)).The references of eligible articles or textbooks were also reviewed to examine other potential sources.

In addition, systematic reviews and validation studies of prediction models were reviewed to identify other relevant articles for our validation study. According to the following inclusion criteria, studies were included if

1. At least one formal prediction model or an update on a previously developed model was presented in the study; and

2. The endpoint was the survival of hepatic failure patients in a study with a cohort design.

Studies using data concerning LT patients who had exact transplant times were excluded. Furthermore, models that used electrocardiogram (ECG) or pulmonary function (PF) data or the depth of ascites as predictor variables were excluded because no reliable substitute variable was available in our longitudinal data.

A primary plan was made to extract necessary information about the models from the original studies. The extracted data involved the name of the model/score, the publication year, the population, the original indication of liver disease, the study design type, the number of centres, the ACLF sample size, the number of HBV cases, participant age, the prediction horizon, the statistical model and the number of predictors.

### Validating the longitudinal study

In total, 232 patients suffering from HBV-ACLF at the Center for Liver Diseases of Nantong Third People’s Hospital, Nantong University between January 2003 and December 2007 were enrolled in the longitudinal cohort: 105(45.3%) patients completed the first 3 months of follow-up, and 83(35.8%) patients completed 5 years of follow-up. The group had a median age of 45(range: 21–69) years, and 77% were male. Baseline information, including the patients’ demographic characteristics, serologic characteristics and therapeutic schemes, was collected from the longitudinal dataset (Table S2). The cumulative risk curves for both 3-month and 5-year were presented in Figure S4.

The study was approved by the institutional review board of Nantong Third People’s Hospital, Nantong University, and the study protocol conformed to the ethical guidelines of the 1975 Declaration of Helsinki. Additionally, written informed consent for inclusion in the study was obtained from each patient (or his or her closest relative).

### Statistical analysis

In the assessment of the validity of the prediction models, the model performance was compared using four key measures, “A, B, C and D”, in terms of the model calibration, discrimination and clinical usefulness^36^.

Calibration reflected the accuracy between the observed endpoints and predictions, which indicated the ability of the model to correctly estimate absolute risks. Calibration could be graphically assessed by the scattered and fitted line as an illustration of the Hosmer-Lemeshow goodness-of-fit test. The parameter alpha (A) was the intercept of the fitted line, which was associated with general calibration and indicated the extent to which predictions were systematically too low or too high^46^. The parameter beta (B) was the calibration slope of the fitted line. A value of B smaller than 1 reflected over-fitting of the model, which could be interpreted as reflecting the need for regression coefficient shrinkage in a prediction model^37^. With an A of 0 and a B of 1, perfect prediction is an ideal line along the 45-degree line^47^.

Discrimination reflected the ability of a model to distinguish a patient with the endpoint (dead) from a patient without the endpoint (alive)^46^. Frequently, the discriminative ability was examined by calculating the corresponding C value or the area value under the receiver operating characteristic (ROC) curve (AUC), in which optimal cut-off values were derived from the sensitivity (true-positive rate) against the 1-specificity (false-positive rate) calculated for consecutive cut-off values for the predicted risk. C ranged from 0 to 1, and values of ≥0.7, ≥0.8 and ≥0.9 were considered to be satisfactory, good and excellent, respectively^48^.

In terms of the model’s clinical usefulness, the net benefit or “benefit score” was determined by adopting a decision-curve analysis (D), which was consistent with the application of an optimal decision threshold to classify patients to balance the likelihood of harm, such as the risk of death and financial costs^38^. The net benefit was calculated using the following formula:


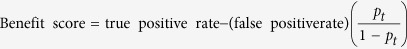


The probability threshold (*p*_t_) produced a relative value for either receiving treatment if the disease was present or avoiding treatment if the disease was not present^49^. The following formula 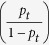 summarized the risk benefit ratio, which was the crucial accessing factor related to the consequence of prognostic models in the establishment of a treatment decision^50^. By applying the “benefit score” on the vertical axis and *p*_t_ on the horizontal axis, decision curves were drawn to graphically assess the value of predictive models.

Stata statistical software (version 13.0; Stata Corp, TX, USA) was adopted to perform all statistical analyses, and statistical significance was defined as *p* ≤ 0.05.

## Additional Information

**How to cite this article**: Shen, Y. *et al.* A Comprehensive validation of HBV related acute-on-chronic liver failure models to assist decision-making in targeted therapeutics. *Sci. Rep.* **6**, 33389; doi: 10.1038/srep33389 (2016).

## Supplementary Material

Supplementary Information

Supplementary Information

Supplementary Information

Supplementary Information

Supplementary Information

Supplementary Information

Supplementary Information

## Figures and Tables

**Table 1:** General characteristics of the models for the prediction of risk among ACHBLF patients included in this study.

Prediction model	Year	Region (Population)	Original indication	Design	No. of centers	Sample size	HBV Cases	No. of predictors	Prediction horizon (months)	Statistical model
MELD (8)	2000	United States	Cirrhosis/TIPS candidates	Retrospective	4	231	—	4	3	Cox
MELD-Na1 (9)	2006	Italy,Austria (82%white, 4%Asian, 5% African American, 9% others)	LT candidates	Prospective	5	753	53	5	6	Cox
MELD-Na2 (10)	2008	United States	Cirrhosis	Prospective	1	6769	—	5	3	Cox
iMELD1 (12)	2007	Italy, Austria	Cirrhosis/TIPS candidates	Retrospective	2	310	—	6	12	Cox
iMELD2 (13)	2014	China	HBV-ACLF	Retrospective	1	220	220	7	3	Cox
MESO (11)	2007	Taiwan, China	Cirrhosis	Retrospective	1	213	125	5	12	Cox
uMELD (14)	2008	United States (73.2% white, 7.7% African American, 3.9% Asian, 14.3% Hispanic, 0.9% others)	LT candidates with normal renal function	Prospective	1	38899	—	3	3	Cox
UKELD (15)	2008	United Kingdom	LT candidates	Prospective	1	1103	—	4	12	Cox
CTP (17)	1973	United Kingdom	Emergency ligation of bleeding oesophageal varices	Prospective	1	38	—	5	6	—
mCTP (18)	2006	Taiwan, China	Cirrhosis	Retrospective	1	436	314	5	6	—
LRM 1 (19)	2009	China	HBV-ACLF	Retrospective	1	204	204	4	3	logistic
LRM 2 (20)	2011	China	HBV-ACLF	Retrospective	1	242	242	6	3	logistic

MELD: model of end-stage liver disease; MELD-Na: sodium MELD; MESO: MELD to sodium ratio; iMELD: integrated MELD; uMELD: updated MELD; UKELD: United Kingdom MELD; CTP: Child-Turcotte-Pugh; mCTP: modified CTP; LRM: logistic regression model; LT: Liver transplant; TIPS: Transjugular intrahepatic portosystemic shunts; HBV-ACLF: hepatitis B virus-related acute-on-chronic liver failure.

**Table 2:** An overview of the four measures (ABCD) of model performance.

SCORES	3-MONTH				5-YEAR			
	A	B	C	D^*^	A	B	C	D^#^
MELD	3.689	0.930	0.651	0.144	0.552	0.983	0.650	0.272
MELD-Na 1	0.689	0.983	0.723	0.194	−1.147	1.010	0.737	0.294
MELD-Na 2	0.731	0.991	0.698	0.191	0.189	0.999	0.708	0.281
iMELD 1	1.154	0.976	0.764	0.214	1.737	0.972	0.800	0.321
iMELD 2	−0.119	1.005	0.745	0.226	−1.319	1.021	0.778	0.337
MESO	1.339	0.973	0.695	0.184	1.703	0.975	0.704	0.281
uMELD	−0.877	1.020	0.769	0.251	−0.964	0.016	0.753	0.369
UKELD	1.429	0.970	0.806	0.261	−0.022	1.002	0.827	0.378
CTP	−0.821	1.013	0.730	0.198	−2.512	1.045	0.778	0.310
mCTP	0.615	0.988	0.737	0.257	−2.823	1.041	0.776	0.377
LRM 1	0.118	0.998	0.759	0.247	1.292	0.979	0.788	0.346
LRM 2	−0.331	1.007	0.817	0.282	0.967	0.985	0.848	0.399

A: calibration constant; B: calibration slope; C: area value under the receiver operating characteristic curve; D^*^: benefit score at *pt* = 40%; D^#^: benefit score at *pt* = 55%; MELD: model of end-stage liver disease; MELD-Na: sodium MELD; MESO: MELD to sodium ratio; iMELD: integrated MELD; uMELD: updated MELD; UKELD: United Kingdom MELD; CTP: Child-Turcotte-Pugh; mCTP: modified CTP; LRM: logistic regression model.
