# Supplementary material for: A comprehensive validation of HBV-related acute-on-chronic liver failure models to assist decision-making in targeted therapeutics
Source: Sci Rep. 2016 Sep 16;6:33389. doi: 10.1038/srep33389 (PMC5025883; doi:10.1038/srep33389)
Supplement: Supplementary Information [file srep33389-s1.pdf]

Comprehensive validation of HBV related acute-on-chronic liver failure models to assist decision-making in targeted therapeutics

Yi Shen,[1] Xulin Wang,[1] Sheng Zhang,[1] Yanmei Liu,[1] Yihua Lu,[1] Feng Liang,[2] Xun Zhuang,[1] & Gang Qin[3]

Y.S., X.L.W and S.Z. contributed equally to this work.

[1]Department of Epidemiology and Medical Statistics, Nantong University, Nantong, China
[2]Qidong Third People's Hospital, Nantong, China
[3]Center for Liver Diseases, Nantong Third People's Hospital, Nantong University, Nantong, China

Table s1 Characteristics of twelve original prediction models

	INR	Creatinine	Bilirubin	Serum sodium	Etiology	age	HE score	HRS	LC	Encephalopathy	Ascites	HBeAg	Albumin
MELD*,#	√	√	√		√								
MELD-Na1*,#	√	√	√	√	√								
MELD-Na2*,#	√	√	√	√	√								
iMELD1*,#	√	√	√	√	√	√							
iMELD2*,#	√	√	√	√	√	√	√						
MESO*,#	√	√	√	√	√								
uMELD*,#	√	√	√										
UKELD*,#	√	√	√	√									
CTP*,#	PT		√							√	√		√
mCTP*,#	PT		√							√	√		√
LRM1*,#	√	√	√		√								
LRM2*,#	PTA					√	√	√	√			√	

*,# $P<0.05$ for hazard ratio at 3-month and 5-year, respectively, Cox proportional hazards model

INR: international normalized ratio; PT: prothrombin time; PTA: prothrombin time activity; HE: hepatic encephalopathy; HRS: Hepatorenal Syndrome; LC: liver cirrhosis; MELD: model of end-stage liver disease; MELD-Na: sodium MELD; MESO: MELD to sodium ratio; iMELD: integrated MELD; uMELD: updated MELD; UKELD: United Kingdom MELD; CTP: Child-Turcotte-Pugh; mCTP: modified CTP; LRM: logistic regression model.

(1) MELD = 11.2×ln(INR)+9.6×ln[Cr(mg/dL)]+3.8×ln[TBIL(mg/dL)]+6.4 [8]

(2) MELD-Na1 = MELD+1.59×(135-Na)， where the minimum value for serum Na is 120 mEq/L and the maximum 135 mEq/L. [9]

(3) MELD-Na2 = MELD-Na-[0.025×MELD×(140-Na)]+140， where the minimum value for serum Na is 125 mEq/L and the maximum 140 mEq/L. [9, 10]

(4) iMELD1 = MELD+(age×0.3)- [Na(mEq/L)×0.7]+100 [12]

(5) iMELD2 = 0.03×age+1.759×(HE score)+0.104×MELD [13]

(6) MESO = [MELD/Na (mEq/l)]×10 [11]

(7) uMELD =1.266ln[1+Cr(mg/dL)]+0.939ln[1+TBIL(mg/dL)]+1.658ln(1+ INR) [14]

(8)UKELD=5.395×lnINR+1.485×lnCr(μmol/)+3.13×lnTBIL(μmol/)-81.565 ×lnNa(mmol/L) +435 [15]

(9) The conventional CTP scoring system is classified from A to C and calculated on the basis of serum bilirubin and albumin levels, the prothrombin time (PT), and the presence and severity of ascites and encephalopathy. [17]

(10)The mCTP scoring system is calculated based on CTP scoring system. An additional 1 point was given for patient whose serum bilirubin level was > 8 mg/dL, PT prolongation > 11 sec, or albumin level < 2.3 g/dL, a modified CTP score of 16-18 indicates severely decompensated cirrhosis and is proposed as CTP class D [18]

(11)LRM1=1.4053+3.6017×HRS+1.2069×LC-1.1555×HBeAg-0.1003×ALB(g/L) -0.042×PTA [19]

(12)LRM2=-1.343+0.772×HE+2.279×HRS+0.85×LC+1.026×HBeAg-2.117 ×PTA/age [20]
