# Supplementary material for: A comprehensive validation of HBV-related acute-on-chronic liver failure models to assist decision-making in targeted therapeutics
Source: Sci Rep. 2016 Sep 16;6:33389. doi: 10.1038/srep33389 (PMC5025883; doi:10.1038/srep33389)
Supplement: Supplementary Information [file srep33389-s2.pdf]

Comprehensive validation of HBV related acute-on-chronic liver failure models to assist decision-making in targeted therapeutics

Yi Shen,[1] Xulin Wang,[1] Sheng Zhang,[1] Yanmei Liu,[1] Yihua Lu,[1] Feng Liang,[2] Xun Zhuang,[1] & Gang Qin[3]

Y.S., X.L.W and S.Z. contributed equally to this work.

[1]Department of Epidemiology and Medical Statistics, Nantong University, Nantong, China

[2]Qidong Third People's Hospital, Nantong, China

[3]Center for Liver Diseases, Nantong Third People's Hospital, Nantong University, Nantong, China

Table s2 Demographic, clinical and laboratory features of the study longitudinal data

Characteristic	Value
Number of patients	232
Male/female	178 (76.7%)/54 (23.3%)
Age (years)	46.1±10.5 (45; 21-69)
HBeAg positivity	142 (61.2%)
HBV DNA (lg copies/mL)	4.1±2.5 (5.0; 0-9.9)
Bilirubin (mg/dL)	22.2±9.2
Creatinine (mg/dL)	0.93±0.74
INR	4.2±2.2
Albumin (g/L)	32.1±5.0
Serum sodium (mEq/L)	132.0±8.9
Preexisting cirrhosis	112 (48.3%)
Ascites	194 (83.6%)
HE	64 (27.6%)
HRS	37 (16.0%)
Diagnostic staging<br>Early/ Mid/ Late	18/63/151
ALSS treatment	104 (44.8%)
Continuous NA treatment	108 (46.6%)
Transplant free survival time (days)	742±840 (100; 3-1825)

Note: Data presented as mean±standard deviation or n (%)

INR: international normalized ratio; HE: hepatic encephalopathy; HRS: Hepatorenal Syndrome; Diagnostic staging: Early, 30%<PTA≤40%; Mid, 20%<PTA≤30%; Late, PTA≤20%; PTA: prothrombin time activity; ALSS: artificial liver support system; NA: nucleos(t)ide analogues.
