# Supplementary material for: A comprehensive validation of HBV-related acute-on-chronic liver failure models to assist decision-making in targeted therapeutics
Source: Sci Rep. 2016 Sep 16;6:33389. doi: 10.1038/srep33389 (PMC5025883; doi:10.1038/srep33389)
Supplement: Supplementary Information [file srep33389-s3.pdf]

Comprehensive validation of HBV related acute-on-chronic liver failure models to assist decision-making in targeted therapeutics

Yi Shen,[1] Xulin Wang,[1] Sheng Zhang,[1] Yanmei Liu,[1] Yihua Lu,[1] Feng Liang,[2] Xun Zhuang,[1] & Gang Qin[3]

Y.S., X.L.W and S.Z. contributed equally to this work.

[1]Department of Epidemiology and Medical Statistics, Nantong University, Nantong, China
[2]Qidong Third People's Hospital, Nantong, China
[3]Center for Liver Diseases, Nantong Third People's Hospital, Nantong University, Nantong, China

Table s3 Sensitivity, specificity, NPV, and PPV for 12 models

Score	3-month						5- year					
	**Cutoff**	**Sensitivity (%)**	**Specificity (%)**	**PPV (%)**	**NPV (%)**	**AUC* (95%CI)**	**Cutoff**	**Sensitivity (%)**	**Specificity (%)**	**PPV (%)**	**NPV (%)**	**AUC# (95%CI)**
MELD	30	54.95	70.25	62.89	62.96	0.65(0.58-0.72)	26	79.87	46.99	73.01	56.52	0.65(0.58-0.72)
MELD-Na 1	41	50.45	87.60	78.87	65.84	0.72(0.66-0.79)	26	89.26	37.35	71.89	65.96	0.74(0.67-0.80)
MELD-Na 2	32	55.86	73.55	65.96	64.49	0.70(0.63-0.77)	26	91.95	32.53	70.62	68.42	0.71(0.64-0.78)
iMELD 1	53	58.56	83.47	76.47	68.71	0.76(0.70-0.82)	43	87.92	50.60	76.16	70.00	0.80(0.74-0.86)
iMELD 2	4.8	63.96	76.86	71.71	69.92	0.75(0.68-0.81)	3.8	93.29	34.94	72.02	74.36	0.78(0.72-0.84)
MESO	2.4	45.95	81.82	69.86	62.26	0.70(0.63-0.76)	1.9	85.91	40.96	72.32	61.82	0.70(0.64-0.77)
uMELD	6.1	78.38	64.46	66.92	76.47	0.77(0.71-0.83)	6	73.83	69.51	81.48	59.38	0.75(0.69-0.82)
UKELD	52	77.48	68.60	69.35	76.85	0.81(0.75-0.86)	49	86.58	59.04	79.14	71.01	0.83(0.77-0.88)
CTP	12	72.07	57.85	61.07	69.31	0.73(0.67-0.79)	11	88.59	54.22	77.65	72.58	0.78(0.72-0.84)
mCTP	14	90.99	48.76	61.96	85.51	0.74(0.67-0.80)	14	86.58	59.04	79.14	71.01	0.78(0.71-0.84)
LRM 1	-2.7	79.28	61.98	65.67	76.53	0.76(0.70-0.82)	-3.5	89.26	46.99	75.14	70.91	0.79(0.73-0.85)
LRM 2	-0.9	81.08	69.42	70.87	80.00	0.82(0.76-0.87)	-1	78.52	77.11	86.03	66.67	0.85(0.80-0.90)

Note: *,# P<0.05 for AUC at 3-month and 5-year, respectively

PPV: positive predictive value; NPV: negative predictive value; CI: confidence interval; AUC: area value under the receiver operating characteristic curve; MELD: model of end-stage liver disease; MELD-Na: sodium MELD; MESO: MELD to sodium ratio; iMELD: integrated MELD; uMELD: updated MELD; UKELD: United Kingdom MELD; CTP: Child-Turcotte-Pugh; mCTP: modified CTP; LRM: logistic regression model.
