# Supplementary figures and images for: A comprehensive validation of HBV-related acute-on-chronic liver failure models to assist decision-making in targeted therapeutics
Source: Sci Rep. 2016 Sep 16;6:33389. doi: 10.1038/srep33389 (PMC5025883; doi:10.1038/srep33389)

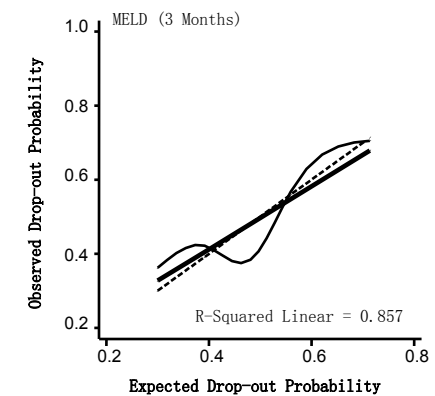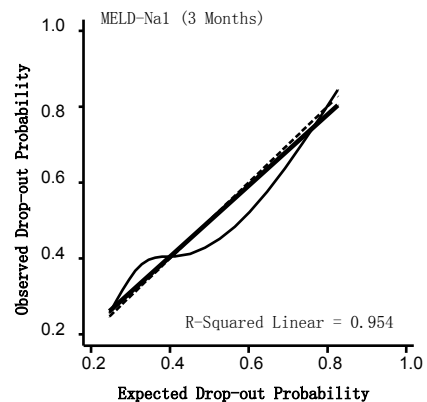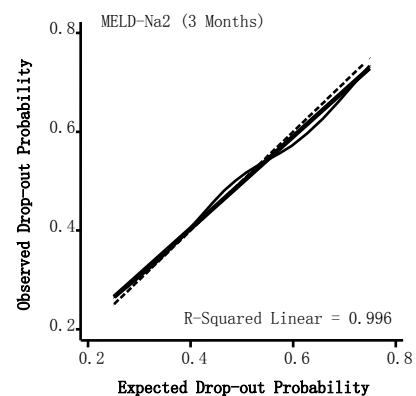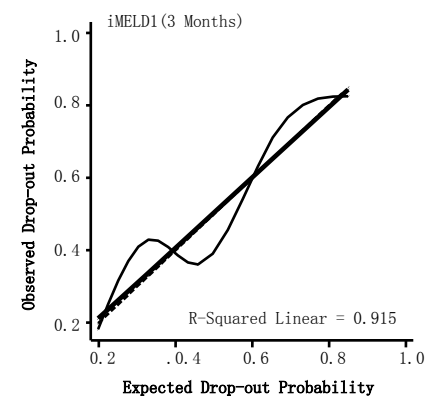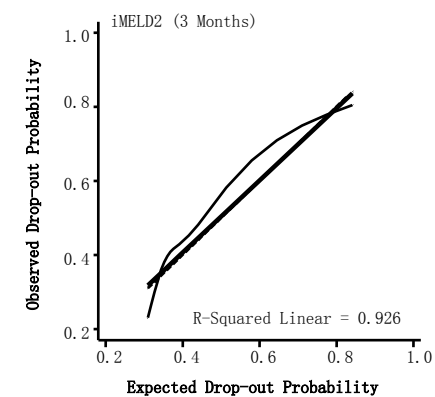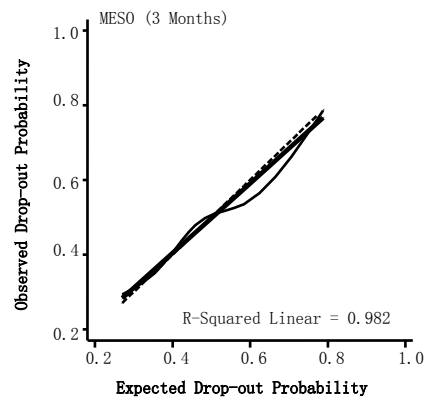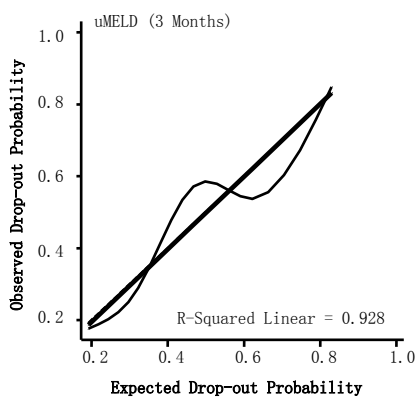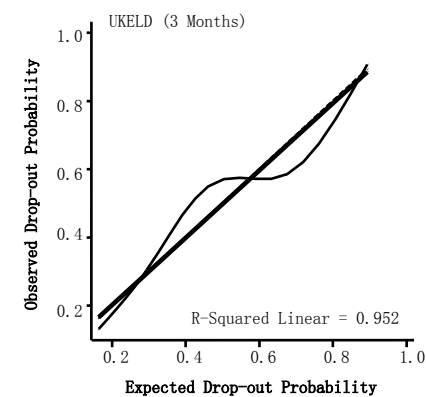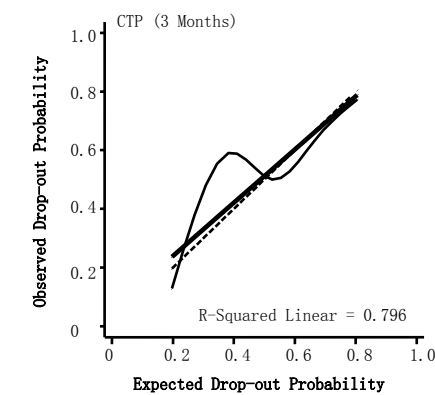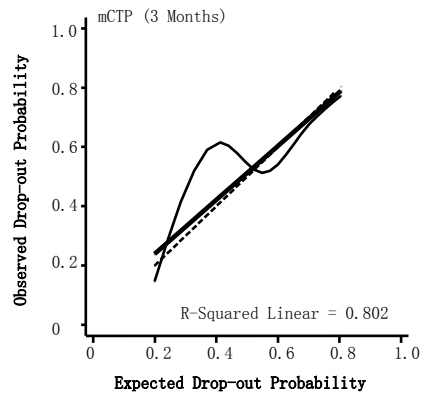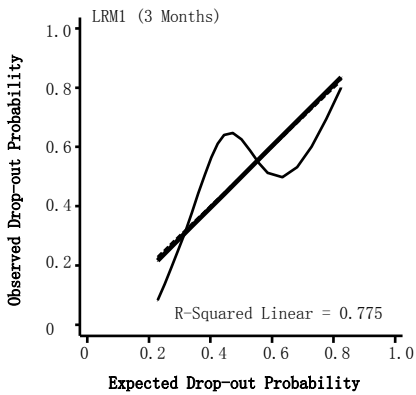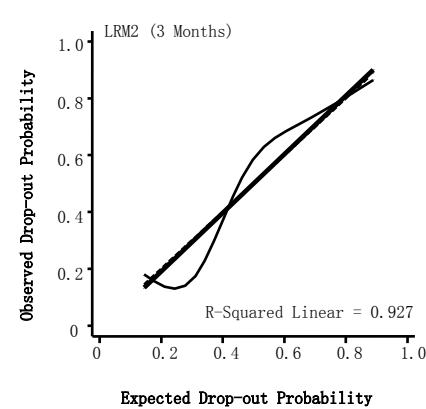

Supplement: Supplementary Information [file srep33389-s4.pdf]

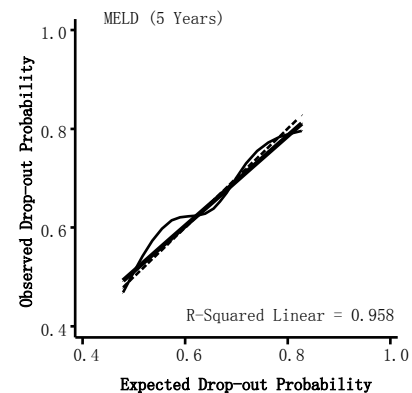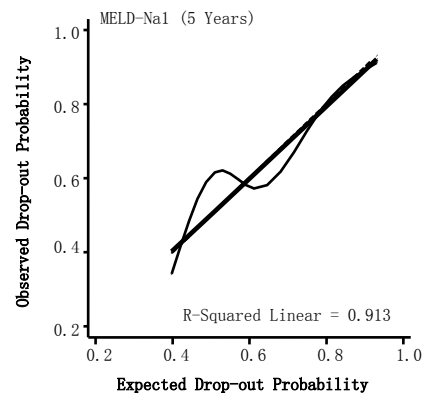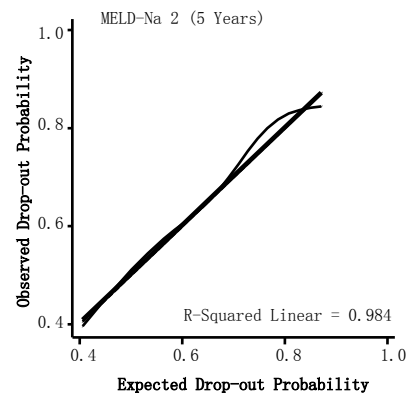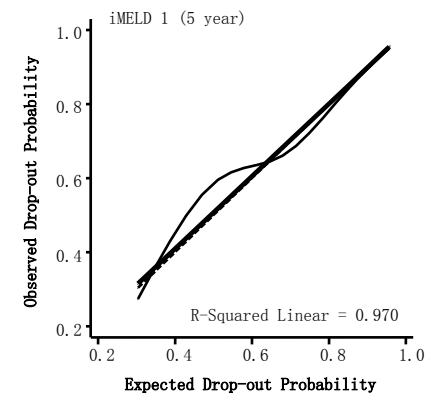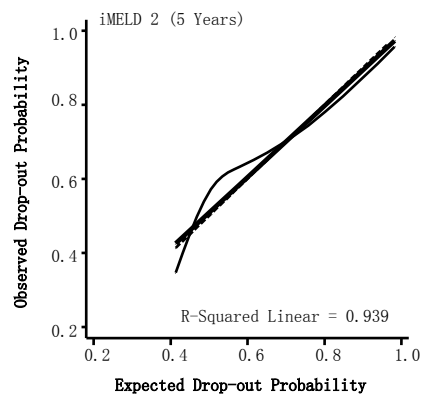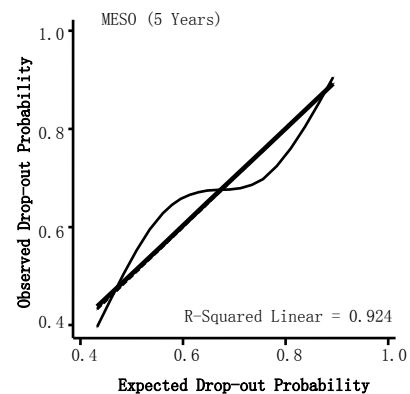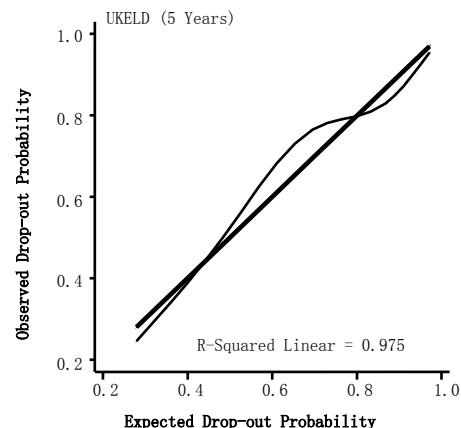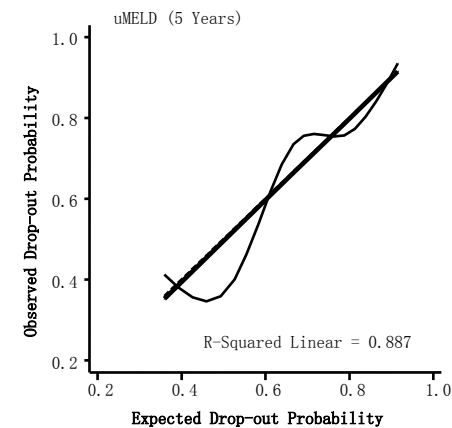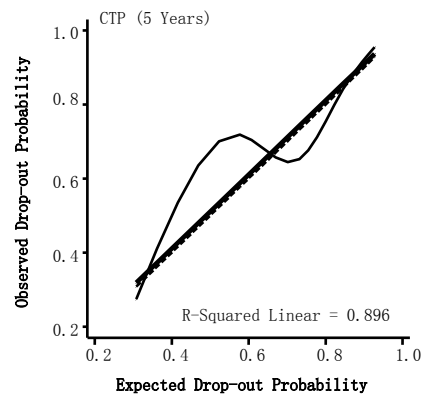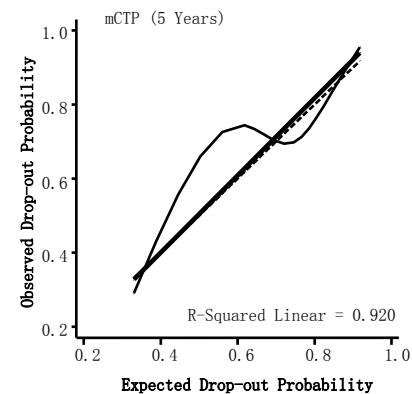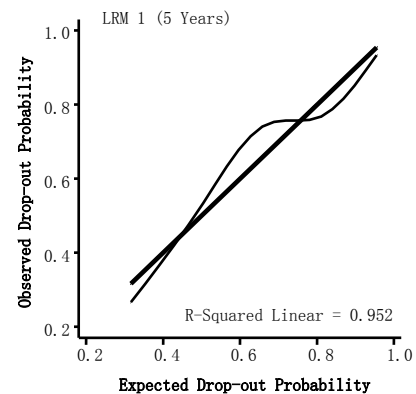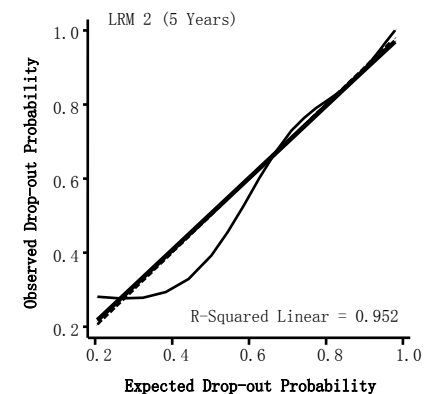

Supplement: Supplementary Information [file srep33389-s5.pdf]

A

90 Days

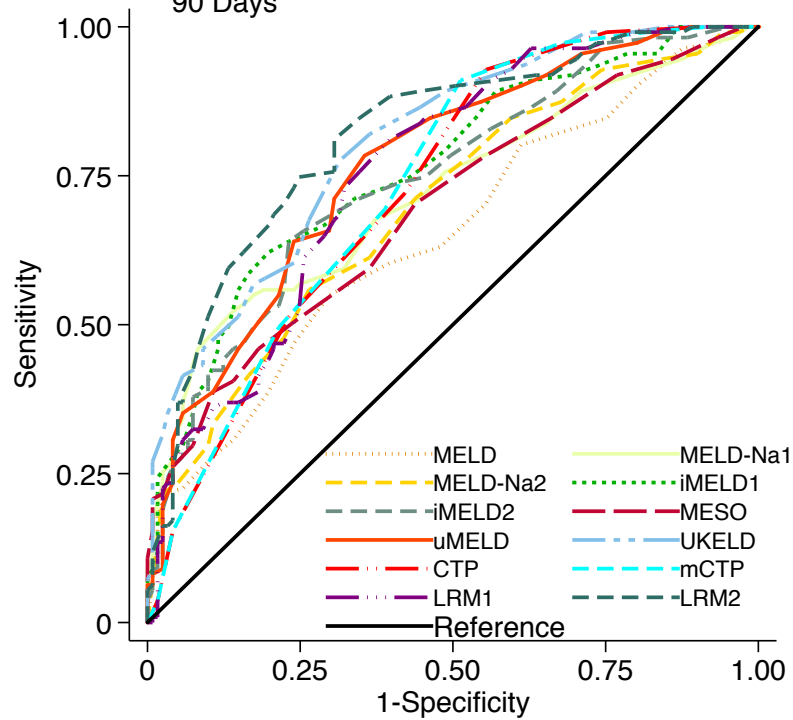

B

5 Years

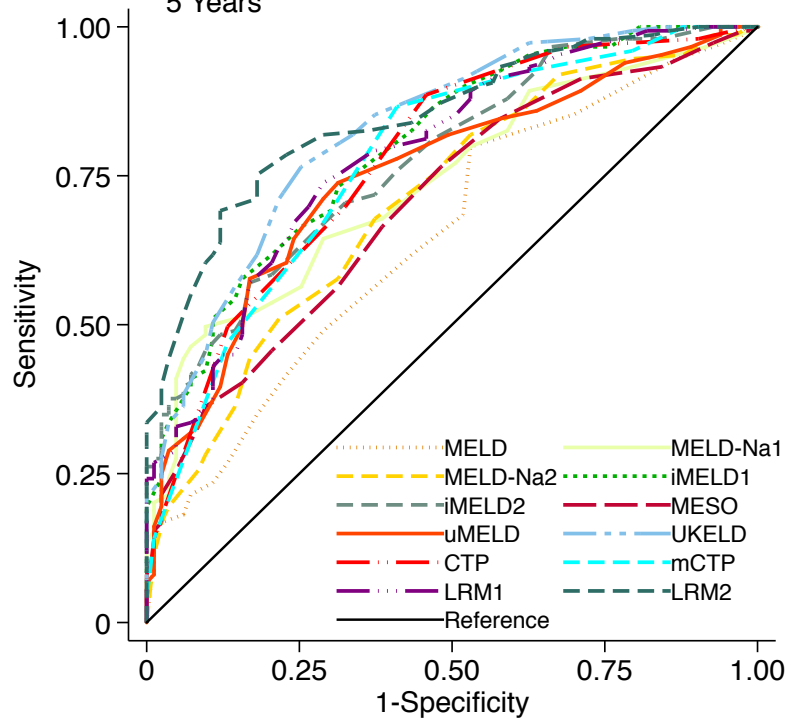

Supplement: Supplementary Information [file srep33389-s6.pdf]

**A**

Nelson-Aalen cumulative hazard estimate

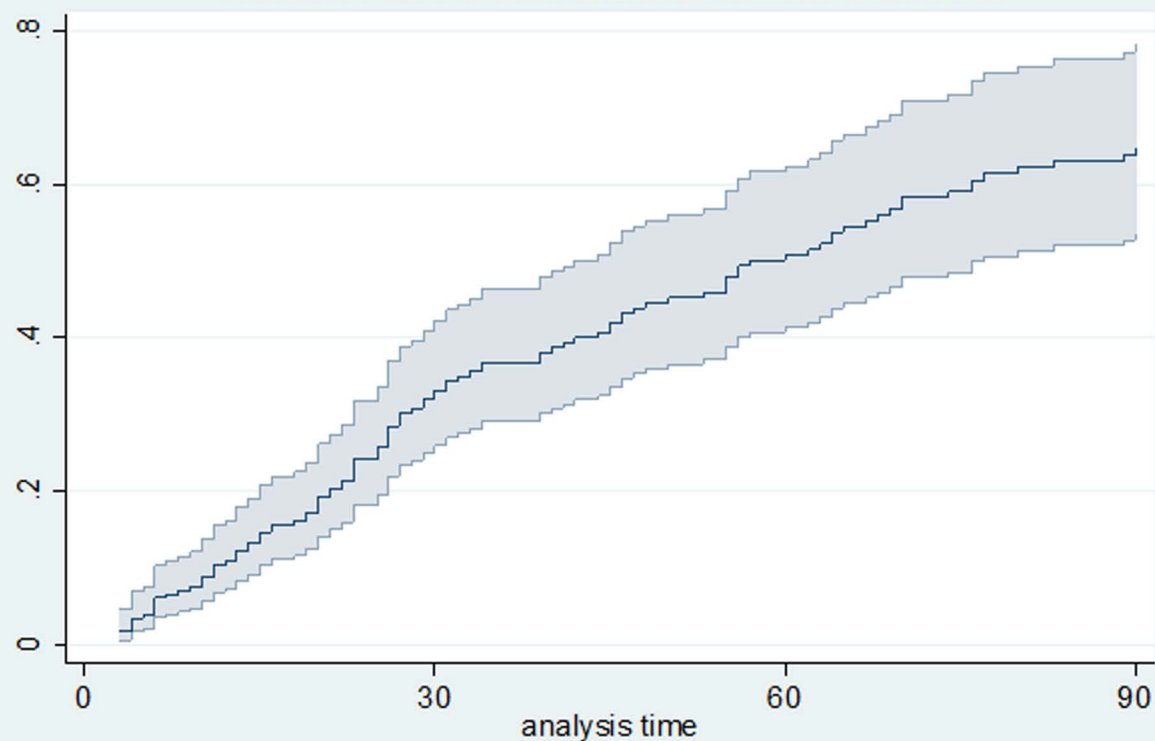**B**

Nelson-Aalen cumulative hazard estimate

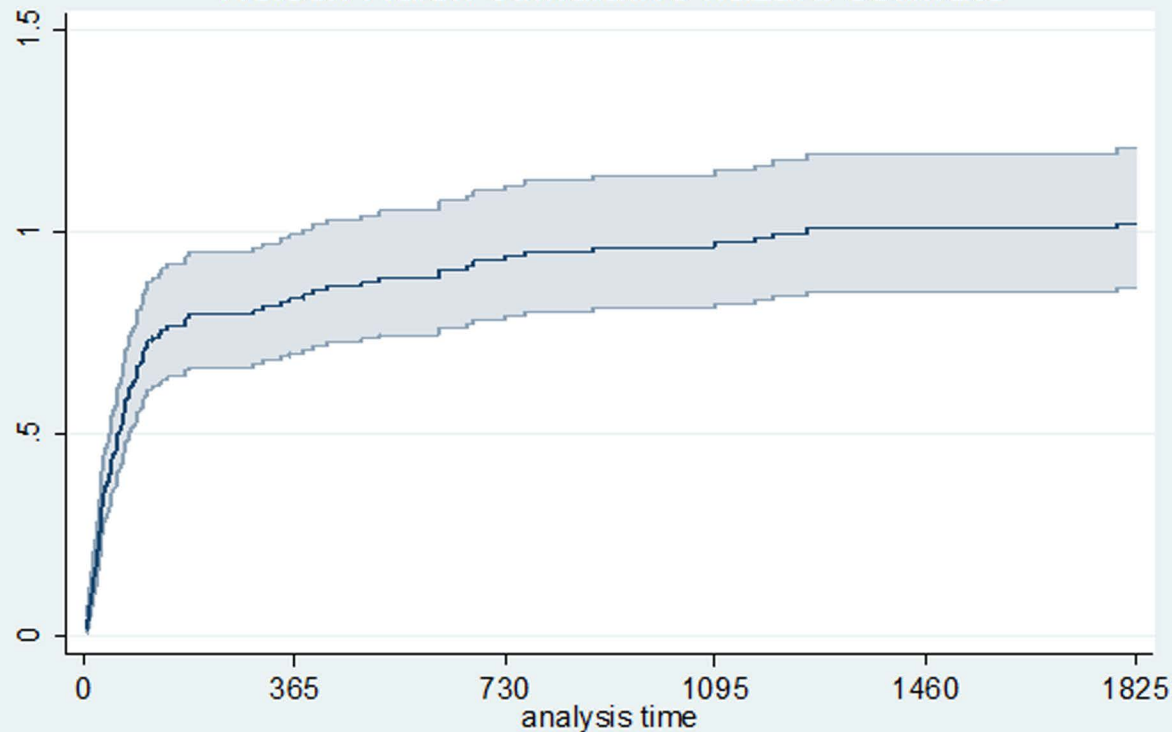

Supplement: Supplementary Information [file srep33389-s7.pdf]
